# Self-actualization and its psychosocial determinants among community health workers in rural Taiwan: a cross-sectional study

**DOI:** 10.1186/s40359-026-04709-w

**Published:** 2026-05-13

**Authors:** Yao-Mei Chuang, Wei-Hsiang Huang

**Affiliations:** 1 Department of Health and Leisure Management, Yuanpei University of Medical Technology, Hsinchu City, Taiwan; 2 Department of Early Childhood Care and Education, University of Kang Ning, Taipei City, Taiwan; 3 Fire Department, New Taipei City Government, No.137, Lane 75, Sec. 3, Kangning Rd., Neihu District, Taipei City, 114311 Taiwan

**Keywords:** Self-actualization, Psychological well-being, Community health workers, Social support, Stress, Burnout, Biopsychosocial model

## Abstract

**Background:**

Community health workers (CHWs) frequently operate in resource-limited rural environments where high work demands and persistent psychosocial stressors may challenge psychological functioning. Guided by the Biopsychosocial Model, this study examined how individual health, social support, life stress, and burnout are associated with self-actualization—conceptualized here as a growth-oriented dimension of positive psychological functioning, which may overlap with broader eudaimonic well-being constructs, rather than as a fully distinct or independent domain.

**Methods:**

A cross-sectional survey was conducted among 328 CHWs employed in Hualien County, Taiwan. Validated instruments were adapted to assess self-rated health, perceived social support, life stress, and burnout components. To avoid construct redundancy with the growth-oriented outcome, the reduced personal accomplishment dimension of burnout was excluded from the primary analysis. Hierarchical multiple regression analyses were performed to examine the incremental associations of biopsychosocial factors with self-actualization while controlling for demographic variables. Given that all variables were collected using self-report measures at a single time point, potential common method variance cannot be ruled out. The 2014 dataset provides a theoretically informative pre-pandemic baseline for understanding relatively stable patterns of psychological functioning in a rural health workforce.

**Results:**

Self-actualization was positively associated with better self-rated health (β = 0.22, *p* < .01) and higher perceived social support (β = 0.28, *p* < .001). Emotional exhaustion (β = –0.15, *p* < .05) and depersonalization (β = –0.20, *p* < .01) were significantly and negatively associated with self-actualization. Life stress also showed a modest but significant negative association (β = –0.15, *p* < .05). The final model explained 39% of the variance in self-actualization, indicating a meaningful level of explained variance, with health and social resources contributing the largest proportion of incremental explanatory power.

**Conclusions:**

The findings indicate that health resources and supportive social environments are closely associated with higher levels of growth-oriented psychological functioning among rural CHWs, whereas psychological strain—particularly emotional and relational depletion—is concurrently associated with lower self-actualization. These findings should be interpreted within a broader eudaimonic well-being framework rather than as evidence of a fully distinct construct. By providing a pre-pandemic reference point, the study offers insight into relatively stable psychosocial mechanisms that may inform future research and intervention design, underscoring the importance of organizational contexts that enhance support and reduce strain to support sustained positive psychological growth in rural community health settings.

**Supplementary Information:**

The online version contains supplementary material available at 10.1186/s40359-026-04709-w.

## Introduction

Guided by the Biopsychosocial Model (BPSM) as the primary theoretical framework (Engel 1977), this study investigates the psychological well-being of healthcare workers (HCWs), which has become an increasingly central topic within occupational and health psychology. Beyond technical competence, HCWs’ motivation, sense of meaning, and perceived capacity for personal growth critically influence their psychological functioning and engagement in demanding work environments (Ryan & Deci 2017; Seligman 2011). Within this BPSM framework, understanding how workers thrive requires moving beyond the mere absence of distress to examine flourishing and eudaimonic functioning (Ryff 2023). While psychological well-being often reflects an individual’s current state of life satisfaction, self-actualization in this study is treated as a growth-oriented dimension of functioning that may overlap with broader eudaimonic well-being constructs, rather than as a fully distinct psychological domain. It is characterized by proactive striving toward fulfilling one’s perceived potential and constructing personal meaning. Rather than treating self-actualization solely as an ultimate developmental endpoint (Maslow 1987), contemporary positive psychology positions it alongside psychological capital as an active process of meaning-making (Waterman 1993). As a specific construct nested within our broader BPSM approach, exploring this growth-oriented dimension is particularly relevant in high-stress professional roles. However, the psychological experiences of community-based HCWs—particularly those working in rural settings—remain insufficiently understood despite mounting evidence that contextual stressors can challenge their well-being.

In Taiwan, community HCWs such as public health nurses, health educators, and outreach personnel work with considerable autonomy across diverse community settings. In rural areas such as Hualien County, the structural characteristics of the environment directly precipitate specific psychosocial mechanisms, notably severe role boundary strain and intensified emotional labor (Laurenzi et al. 2021). These workers often reside within the same small, close-knit communities they serve, resulting in blurred boundaries between professional responsibilities and private life. They face unique geographical challenges, including navigating mountainous terrain to provide home-based care, frequently with limited organizational support or clinical resources. While regional demographic data from the study period (Hualien County Government, 2014) indicate a markedly high Indigenous (exceeding 95% in certain townships) and aging population, the primary theoretical relevance of these demographics lies in the culturally sensitive chronic disease management and complex social welfare coordination they necessitate. These factors may contribute to increased occupational strain experienced by frontline workers. Prior research suggests that sustained exposure to emotionally demanding work conditions may heighten perceived stress, reduce psychological well-being, and erode intrinsic motivation (Cherniack et al. 2024; Ruotsalainen et al. 2015). For frontline rural HCWs operating under these conditions, self-actualization may function not merely as a philosophical ideal, but as a potentially important psychological resource that supports engagement, meaning-making, and sustained functioning in resource-constrained environments.

Hualien County, a predominantly rural region, exemplifies these challenges. HCWs in this setting frequently report heavy workloads, insufficient support systems, and limited access to mental health resources. To clarify the conceptual boundaries within our framework, we treat life stress and burnout as distinct constructs (Maslach & Leiter 2016). Life stress is conceptualized here as a broad *environmental strain*, capturing both the occupational demands and the socio-environmental pressures inherent in rural living. Burnout, conversely, is treated as a severe psychological consequence of chronic resource depletion. In the present study, burnout is conceptualized primarily as an indicator of chronic psychological strain rather than as a comprehensive outcome construct. Accordingly, we focus on emotional exhaustion and depersonalization as experiential dimensions most directly relevant to motivation and meaning-related processes. Reduced personal accomplishment is not disregarded conceptually; rather, it is not included analytically in order to minimize conceptual redundancy with growth-oriented outcome measures. These sustained pressures may influence workers’ perceived capacity for personal development and meaning-making, core elements of self-actualization (Burke et al. 2022).

Although previous studies have examined stress, burnout, or quality of life among clinical health workers (Khatatbeh et al. 2022), limited research has explored how multiple psychosocial determinants jointly relate to self-actualization in community-based HCWs. In this study, self-actualization is operationalized through the lens of perceived personal growth and life integration, as measured by the Health Promoting Lifestyle Profile (HPLP). Within the theoretical framework of the HPLP, the self-actualization subscale (often conceptualized alongside spiritual growth in later iterations) was originally designed to capture the development of inner resources, proactive meaning-making, and the maximization of human potential, rather than merely reflecting physical health behaviors (Pender et al. 2015; Walker et al. 1987)**.** However, it should be acknowledged that several elements of the HPLP-based operationalization (e.g., meaning in life, personal growth) are conceptually proximal to eudaimonic well-being, and therefore the observed associations may partially reflect shared variance within a broader growth-oriented well-being domain rather than entirely distinct constructs. We explicitly acknowledge that this operationalization reflects subjective growth-oriented functioning and aligns closely with contemporary constructs of eudaimonic well-being, rather than Maslow’s full hierarchical conception of self-actualization as an ultimate developmental endpoint. Conceptually, self-actualization is treated not simply as a proxy for resilience or global well-being, but as a construct that partially overlaps with broader eudaimonic functioning while emphasizing individuals’ perceived potential and pursuit of personally meaningful goals (Wong 2011). Examining its psychosocial correlates may therefore provide valuable psychological insight into how HCWs function under sustained environmental and emotional demands, without implying a comprehensive assessment of self-actualization as a final developmental state.

Returning to the BPSM**,** this study examines how biological factors (self-rated health), psychological factors (life stress and burnout), and social factors (perceived social support) relate to self-actualization among rural HCWs. Although stress and burnout are conceptually linked, they are treated as parallel correlates rather than as components of a mediational pathway, consistent with the cross-sectional design and the study’s emphasis on mapping concurrent psychosocial associations. The BPSM offers a holistic framework for understanding how growth-oriented psychological functioning emerges from the interaction of individual and contextual influences.

Using Hualien County as a case context, this study aims to: (1) describe the psychological and health characteristics of community HCWs; (2) examine associative relationships among health resources, stress, burnout, social support, and self-actualization; and (3) identify implications for supporting psychological well-being in resource-limited community health settings.

By situating these findings within a pre-digitalization and pre-pandemic context, the present study provides a psychological baseline that clarifies how multiple psychosocial determinants were jointly associated with positive psychological functioning prior to major systemic shifts in healthcare delivery. Notably, subsequent policy developments in Taiwan, such as the implementation of Long-Term Care 2.0, have reshaped the structural demands placed on community health workers, highlighting the importance of interpreting these baseline findings within an evolving policy context. This historical anchor is not merely descriptive; it may contribute to theory by offering a reference point for examining psychosocial mechanisms—such as role boundary strain and social support—prior to the influence of recent global health crises. As contemporary scholars emphasize, understanding how changing historical conditions shape psychological functioning is critical for the future study of well-being (Ryff et al. 2021). Therefore, mapping these stable, pre-crisis baseline relationships may provide a useful foundation for researchers to design sustainable, long-term interventions that address structural rural health challenges, rather than merely reacting to pandemic-induced trauma.

## Methods

### Study design and framework

This study employed a cross-sectional design conducted between September 2014 and January 2015 to examine psychological and health-related correlates of self-actualization among community-based healthcare workers (HCWs) in Hualien County, Taiwan.

Consistent with the study’s focus on positive psychological functioning, self-actualization was operationalized in this study as a growth-oriented dimension of functioning reflecting perceived personal development and meaning-making. Although conceptually related to broader eudaimonic well-being, it was treated here as a more specific indicator of perceived growth-oriented functioning rather than as a comprehensive indicator of overall well-being. Self-actualization reflects individuals’ perceived capacity to realize their potential and achieve personal meaning, consistent with foundational theories of human motivation and flourishing (Maslow 1987; Seligman 2011; Wong 2011).

Independent variables included demographic characteristics, biological and psychological resources (Individual Health and Psychological Well-being), social-contextual resources (Social Support and Environmental Satisfaction), life stress, and two core burnout dimensions—emotional exhaustion and depersonalization. These variables were selected based on their theoretical relevance to psychological functioning and well-being (Maslach & Leiter 2016). The reduced personal accomplishment dimension was conceptually acknowledged but was not included in the regression models in order to avoid construct redundancy with the growth-oriented outcome of self-actualization.

Guided by the Biopsychosocial Model (BPSM), the study examined how biological, psychological, and social determinants collectively relate to self-actualization. BPSM provides a holistic framework for understanding human functioning as shaped by interacting individual and contextual influences (Engel 1977). In the present study, the BPSM served as an organizing and interpretive framework rather than a causal or mediational model. To evaluate the incremental contribution of each conceptual domain, hierarchical multiple regression was performed, entering predictors in sequential blocks: demographic factors, health indicators, social-contextual factors, and stress- and burnout-related variables. Hierarchical regression is widely used to assess stepwise explanatory value across theoretically meaningful blocks in psychological research (Cohen et al. 2003).

Hualien County is administratively classified as a rural and sparsely populated region, characterized by limited healthcare infrastructure and demanding work conditions. Although the study does not adopt a specific international rural classification system, the demographic and geographic characteristics of Hualien are broadly comparable to rural regions described in global health and psychology literature. This contextualization is intended to clarify the occupational setting of the participants rather than to support statistical generalization beyond comparable rural health systems.

### Participants and sample size estimation

#### Participants

Eligible participants were community-based healthcare workers employed by the Hualien County Health Bureau and its affiliated community health centers during the study period. Participants included personnel directly involved in community health services, such as public health nursing, health education, outreach services, and related frontline public health duties. A total of 360 eligible workers were invited to participate, and 328 valid questionnaires were returned and included in the final analysis.

#### Exclusion criteria

Individuals on extended leave (e.g., parental or medical leave), external contractors without direct involvement in community health services, and staff who declined participation or did not complete the questionnaire were excluded from the analysis.

#### Sampling method

A total population sampling approach was used: all eligible personnel within the Hualien County Health Bureau and its community health centers were invited to participate. Paper-based questionnaires were distributed and collected through coordination with administrative units.

Of the 360 eligible individuals, 328 returned valid questionnaires. The remaining 32 individuals either declined participation, were on extended leave, or returned incomplete questionnaires. Because organizational distribution procedures were used, participation may have been influenced by workplace context, and nonresponse bias cannot be ruled out. Individuals who did not participate may have differed systematically from respondents in ways not captured by the available data. Therefore, generalizability should be limited to similar institutional and rural public health settings.

#### Sample size estimation

Sample size estimation for the hierarchical regression analysis was performed using G*Power 3.1 (Faul et al. 2009). Assuming a medium effect size (f^2^ = 0.15), α = 0.05, and a power level of 0.80, the required sample size was calculated based on testing the full regression model comprising 15 predictors, rather than the incremental ΔR^2^ of a specific block. Under these assumptions, the minimum required sample size was 139 participants.

In addition to power analysis, methodological guidelines for questionnaire-based research recommend determining sample size based on the number of items in the largest subscale. According to established recommendations, adequate sample size should be at least 5–10 times the number of measured items (Hair et al. 2010). The largest subscale in this study—Individual Health and Psychological Well-being—contained 13 items, indicating a minimum recommended sample of 65–130 participants.

Furthermore, regression-specific rules of thumb suggest that adequate sample size should meet either N ≥ 50 + 8k for testing the overall model or N ≥ 104 + k for testing individual predictors (Green 1991). With k representing the number of predictors entered in the final regression model, the achieved sample size of 328 far exceeded all recommended thresholds. Taken together, these criteria indicate that the achieved sample size was sufficient to support stable estimation of the hierarchical regression models.

### Research instruments

A structured questionnaire was administered to assess background characteristics and a range of psychological and health-related factors among community healthcare workers. The instrument comprised two major components: background information and validated measures reflecting individual health, psychological well-being, social resources, stress, and burnout.

Background information included age, gender, education level, marital status, number of children, job role, workplace setting, and years of service. Among these variables, age group, job role, workplace setting, years of service, and employment type were retained as covariates in the regression analyses based on their theoretical relevance in prior occupational health research and their empirical association with the outcome in preliminary analyses, rather than on statistical screening alone (Khatatbeh et al. 2022; Park & June 2022).

Psychological and health-related constructs were measured using selected subscales from the Chinese WHOQOL-BREF (Kalfoss et al. 2021), the Life Stress Questionnaire CHQ-12 (Zhang et al. 2020), the Copenhagen Burnout Inventory (Moser et al. 2023), and the self-actualization subdimension of the Health-Promoting Lifestyle Profile (Chen et al. 1997).

The questionnaire was adapted from established instruments through a multi-step process. First, items were selected based on conceptual alignment with the study constructs and to minimize redundancy across related domains. Second, because Chinese versions of the source instruments were available, item wording was reviewed and harmonized for semantic consistency and contextual appropriateness for rural Taiwanese HCWs. Third, a panel of five academic and clinical experts (including specialists in public health, nursing, health education, and community healthcare practice) reviewed the initial item pool for content relevance, clarity, and contextual suitability. Finally, a pilot test was conducted with 30 community health workers to assess readability and conceptual comprehensibility prior to formal administration. The detailed items of the English version of the questionnaire used in this study are provided in Supplementary File 1. Only domains theoretically relevant to health, social support, stress, burnout, and self-actualization were included in order to reduce respondent burden and to avoid construct redundancy across conceptually related measures.

Prior to factor analysis, item discrimination and item–total correlations were examined, and items with inadequate psychometric performance were removed. Item refinement procedures are summarized in Table 1, with detailed results presented in Tables 2 and 3. Exploratory factor analysis with promax rotation was conducted to examine the structural coherence of the retained items.

**Table 1:** Summary of questionnaire revision process and reliability/validity results for each dimension

Questionnaire	Dimension Name	Original Items	Items Removed in Item Analysis	Items Removed in EFA	Retained Items	Cronbach’s α
WHOQOL	Individual Health and Psychological Well-being	28	3	3	13	.91
	Social Support and Environmental Satisfaction				9	.86
CHQ-12	Life Stress	12	2	0	10	.87
CBI	Emotional Exhaustion	21	2	0	9	.94
	Depersonalization				6	.91
	Reduced Personal Accomplishment				4	.87
HPLP	Self-actualization	8	0	0	8	.95

Results are based on item analysis and exploratory factor analysis (EFA). Retained items met criteria for critical ratio (*p* <.001), item-total correlation (R > 0.3), and Cronbach’s α > 0.8 for each dimension. EFA utilized oblique rotation, and factor loadings > 0.4 were retained

**Table 2:** Summary of questionnaire item analysis (*N* = 328)

WHOQOL-BREF				CHQ-12				CBI				HPLP			
Items	Critical Ratio	Homogeneity test (total score α =.886)		Items	Critical Ratio	Homogeneity test (total score α =.827)		Items	Critical Ratio	Homogeneity test (total score α =.923)		Items	Critical Ratio	Homogeneity test (total score α =.946)	
		Item-Total Correlation (R)	α (if item deleted)			Item-Total Correlation (R)	α (if item deleted)			Item-Total Correlation (R)	α (if item deleted)			Item-Total Correlation (R)	α (if item deleted)
WH1	12.51***	0.62	0.88	C1	17.14***	0.73	0.80	CB1	14.20***	0.67	0.92	H1	20.18***	0.82	0.94
WH2	12.24***	0.60	0.88	C2	14.62***	0.71	0.80	CB2	13.92***	0.73	0.92	H2	32.67***	0.90	0.94
WH3	-0.48	-0.03	0.90	C3	16.70***	0.77	0.80	CB3	16.37***	0.76	0.92	H3	28.61***	0.90	0.94
WH4	-3.47	-0.18	0.90	C4	5.91***	0.54	0.84	CB4	15.54***	0.77	0.92	H4	17.00***	0.78	0.94
WH5	14.53***	0.69	0.88	C5	15.20***	0.71	0.80	CB5	13.23***	0.72	0.92	H5	21.03***	0.82	0.94
WH6	14.78***	0.71	0.88	C6	16.91***	0.71	0.80	CB6	17.59***	0.80	0.92	H6	22.90***	0.86	0.94
WH7	12.79***	0.65	0.88	C7	7.12***	0.35	0.84	CB7	15.80***	0.77	0.92	H7	25.91***	0.87	0.94
WH8	13.09***	0.68	0.88	C8	15.55***	0.69	0.81	CB8	6.95***	0.50	0.93	H8	25.11***	0.87	0.94
WH9	9.31***	0.51	0.88	C9	14.75***	0.71	0.80	CB9	13.83***	0.79	0.92				
WH10	14.85***	0.70	0.88	C10	1.60	0.11	0.85	CB10	11.98***	0.73	0.92				
WH11	13.95***	0.66	0.88	C11	13.74***	0.65	0.81	CB11	9.99***	0.68	0.92				
WH12	10.63***	0.57	0.88	C12	11.36***	0.59	0.81	CB12	9.68***	0.68	0.92				
WH13	13.41***	0.32	0.90					CB13	9.02***	0.66	0.92				
WH14	10.88***	0.64	0.88					CB14	8.68***	0.65	0.92				
WH15	12.68***	0.61	0.88					CB15	10.88***	0.66	0.92				
WH16	13.60***	0.62	0.88					CB16	9.49***	0.63	0.92				
WH17	12.57***	0.69	0.88					CB17	7.87***	0.50	0.92				
WH18	14.34***	0.65	0.88					CB18	10.94***	0.62	0.92				
WH19	12.62***	0.72	0.88					CB19	10.15***	0.63	0.92				
WH20	10.18***	0.65	0.88					CB20	8.39***	0.52	0.92				
WH21	11.36***	0.57	0.88					CB21	1.17	0.13	0.93				
WH22	10.01***	0.62	0.88												
WH23	11.02***	0.55	0.88												
WH24	10.12***	0.61	0.88												
WH25	10.12***	0.57	0.88												
WH26	-7.36	-0.44	0.90												
WH27	12.27***	0.60	0.88												
WH28	8.66***	0.53	0.88												

Results from item analysis include critical ratio, item-total correlation, and Cronbach’s α for retained items within each questionnaire dimension. Items failing to meet thresholds for critical ratio (*p* <.001), correlation (R > 0.3), or reliability improvement (α increases upon deletion) were excluded

**Table 3:** Summary of factor analysis for the questionnaire (*N* = 328)

Item/Original Item	WHOQOL-BREF		Copenhagen Burnout Inventory (CBI)			
	Individual Health and Psychological Well-being	Social Support and Environmental Satisfaction	Item/Original Item	Emotional Exhaustion	Depersonalization	Reduced Personal Accomplishment
WP1/WH1	.90		WE1/CB4	.93		
WP2/WH2	.89		WE2/CB3	.92		
WP3/WH10	.83		WE3/CB2	.89		
WP/WH16	.75		WE4/CB5	.88		
WP5/WH8	.75		WE5/CB1	.87		
WP6/WH7	.65		WE6/CB6	.80		
WP7/WH15	.54		WE7/CB9	.63		
WP8/WH5	.53		WE8/CB10	.57		
WP9/WH17	.49		WE9/CB7	.55		
WP10/WH9	.49		WD1/CB13		.94	
WP11/WH14	.47		WD2/CB14		.94	
WP12/WH6	.44		WD3/CB12		.92	
WP13/WH11	.43		WD4/CB16		.84	
WS1/WH22		.91	WD5/CB11		.72	
WS2/WH23		.76	WD6/CB15		.64	
WS3/WH24		.74	WL1/CB18			.92
WS4/WH20		.67	WL2/CB17			.87
WS5/WH19		.64	WL3/CB19			.83
WS6/WH28		.61	WL4/CB20			.77
WS7/WH18		.57				
WS8/WH25		.56				
WS9/WH21		.54				
Eigenvalue	9.26	1.60		9.13	2.20	2.12
Number of Items Retained	13	9		9	6	4
Explained Variance (%)	42.11	7.28		48.05	11.59	11.13
Cumulative Explained Variance (%)	42.11	49.39		48.05	59.63	70.76
Sampling Adequacy (KMO)	0.93			0.93		
Bartlett’s Test of Sphericity χ2	3683.29***			4739.96***		

Exploratory factor analysis (EFA) results are based on eigenvalues > 1 and supported by parallel analysis. Factors were extracted using oblique rotation (KMO = 0.93, Bartlett’s test of sphericity χ² significant at *p* <.001). Factor loadings > 0.4 were retained, with cumulative variance explained approaching or exceeding 50% across the adapted measures

Because the instruments were adapted from established measures and applied to a specific rural healthcare context, exploratory factor analysis (EFA) was employed to examine the appropriateness and structural coherence of the adapted item set. However, confirmatory factor analysis (CFA) would provide stronger evidence of structural validity, and the absence of CFA should be considered a limitation of the present study. The primary aim of EFA in this study was to assess structural coherence and to detect potential construct redundancy among adapted items, rather than to test or confirm a strictly specified a priori measurement model. Factor retention was guided by eigenvalues, scree plots, and parallel analysis. Items with insufficient loadings or cross-loadings were excluded.

This process produced a two-factor structure for the WHOQOL-BREF—Individual Health and Psychological Well-being (13 items) and Social Support and Environmental Satisfaction (9 items)—with Cronbach’s α values of 0.91 and 0.86. Although this data-driven restructuring deviated from the original WHOQOL-BREF domain structure, the resulting two-factor solution showed acceptable internal consistency and interpretive coherence in this sample. Nevertheless, these revised dimensions should not be assumed to be fully equivalent to the original WHOQOL-BREF constructs, and their interpretation should therefore remain cautious. The refined CHQ-12 contained ten items (α = 0.87). For burnout, the emotional exhaustion (9 items), depersonalization (6 items), and reduced personal accomplishment (4 items) subscales showed reliability coefficients of 0.94, 0.91, and 0.87, respectively. The eight-item self-actualization subscale demonstrated excellent internal consistency (α = 0.95). Although reduced personal accomplishment was retained for reliability reporting, it was not included in subsequent regression analyses to avoid construct redundancy with the growth-oriented outcome measure.

Comparisons with reliabilities reported in the original validation studies confirmed that the adapted scales maintained acceptable psychometric properties in this population.

### Statistical analysis

All analyses were performed using IBM SPSS Statistics version 22. Descriptive statistics—including means, standard deviations, frequencies, and percentages—were used to summarize demographic characteristics and major study variables.

Exploratory factor analysis (EFA) with promax rotation was applied to evaluate the structural validity of the adapted instruments. Decisions regarding factor retention were guided by eigenvalues, scree plots, and parallel analysis, consistent with established recommendations for EFA (Bae & Hong 2024). Internal consistency was examined using Cronbach’s α, following conventional thresholds for reliability (Thorndike 1995).

Because all variables were collected using self-report questionnaires, potential common method variance (CMV) was assessed. Harman’s single-factor test was conducted by entering all measurement items into an unrotated exploratory factor analysis. The largest single factor accounted for less than 50% of the total variance, consistent with recommendations for evaluating common method bias (Aguirre-Urreta & Hu, 2019). However, this procedure alone is insufficient to rule out common method variance, and given the single-source, cross-sectional design, the observed associations may have been partially inflated by shared method variance. The findings should therefore be interpreted with appropriate caution.

For descriptive and comparative purposes, age and years of service were categorized into meaningful groups that reflect typical career stages and experience levels within the public health workforce. These groupings enhanced interpretability in group comparisons; for regression analyses, dummy-coded variables were used.

Pearson correlation coefficients were calculated to examine associations among self-actualization, health indicators, social support, life stress, and burnout. Independent-sample t-tests and one-way ANOVA were conducted to compare self-actualization across demographic subgroups.

Hierarchical multiple regression analysis was employed to assess the incremental contribution of each domain of predictors to self-actualization. Predictor blocks were entered sequentially—demographic variables, health indicators, social-contextual variables, life stress, and burnout dimensions—following recommended practices for evaluating stepwise explanatory value in behavioral research (Cohen et al. 2003). Regression coefficients were interpreted as concurrent associations rather than directional or causal effects, consistent with the cross-sectional design of the study.

Prior to interpreting the regression models, diagnostic tests were conducted to confirm core regression assumptions. Variance inflation factors (VIFs) were examined to assess multicollinearity, with all values ranging between 1.18 and 3.25, well below the conservative threshold of 5. Furthermore, residual diagnostics were systematically reviewed: scatterplots of standardized residuals against predicted values confirmed homoscedasticity and linearity, while normal P–P plots of regression standardized residuals supported the normality of error terms (Field 2024). No substantial violations of regression assumptions were detected.

Interaction terms (e.g., life stress × social support) were not examined, as the present study focused on main-effect associations among biopsychosocial variables. Mediation analyses were not conducted because the cross-sectional design does not permit temporal ordering of variables, and the analysis was not intended to test causal or indirect pathways. Future research may explore moderation or mediation effects using longitudinal or experimental designs.

## Results

### Participant characteristics: analysis of background variables

A total of 328 community healthcare workers participated in the study. The majority of participants were female (77.4%) and aged between 31 and 50 years (63.7%). Nearly half held an associate or bachelor’s degree (43.3%), and 72.6% were married. Most participants occupied non-supervisory positions (90.5%) and were stationed in community health centers (58.8%). Years of service were broadly distributed across categories, and full-time employees comprised 59.8% of the sample. Overall, the sample primarily consisted of middle-aged frontline workers with stable employment and educational backgrounds.

### Self-actualization and performance in related dimensions

Mean scores and standard deviations for all major constructs are presented in Table 4. Given the absence of established clinical cutoffs or normative benchmarks for these contextually adapted instruments, descriptive score levels (e.g., "moderate" or "moderately high") were tentatively classified based on their relative position to the theoretical midpoint of each scale's possible score range and should therefore be interpreted with caution.

**Table 4:** Descriptive statistics for self-actualization and related dimensions (*N* = 328)

Dimension	No. of Items	Possible Score Range	Scale Total Score, Mean ± SD	Highest-Scoring Item (Item Mean ± SD)
Individual Health and Psychological Well-being	13	13—65	44.15 ± 7.75	WP11(3.92 ± 0.85)
Social Support and Environmental Satisfaction	9	9—45	32.90 ± 4.50	WS5 (3.77 ± 0.67)
Life Stress	10	10—40	17.98 ± 5.73	C5 (2.05 ± 0.84)
Emotional Exhaustion	9	9—36	20.25 ± 5.28	WE2 (2.63 ± 0.74)
Depersonalization	6	6—24	12.13 ± 3.06	WD5 (2.13 ± 0.66)
Reduced Personal Accomplishment	4	4—16	10.06 ± 2.49	WL4 (2.59 ± 0.76)
Self-actualization	8	8—32	22.86 ± 5.25	H5 (3.07 ± 0.72)

Scale scores are reported as the mean of summed item scores for each construct (possible score ranges are provided)

*Highest-scoring item* refers to the single item within each construct that demonstrated the highest mean score across participants; item-level means and standard deviations are reported for descriptive illustration only

Participants reported moderate levels of individual health and psychological well-being (M = 44.15, SD = 7.75). The highest-scoring items reflected opportunities for leisure activities, whereas lower scores were observed for items related to the impact of physical discomfort on daily functioning. Social support and environmental satisfaction also showed moderately high levels (M = 32.90, SD = 4.50), with satisfaction with oneself receiving the highest ratings.

The mean score for life stress was 17.98 (SD = 5.73), with sleep difficulties emerging as the most prominent stress-related symptom. Regarding burnout, emotional exhaustion (M = 20.25, SD = 5.28) and depersonalization (M = 12.13, SD = 3.06) indicated moderate levels of psychological strain.

Self-actualization demonstrated a mean score of 22.86 (SD = 5.25), suggesting mid-range levels of perceived personal growth and meaning-oriented functioning among participants.

### Differences across background variables

One-way analyses of variance (ANOVA) were conducted to examine differences in self-actualization across selected background characteristics. Significant differences in self-actualization were observed across age groups (F = 4.68, *p* = 0.003), job role (F = 8.74, *p* = 0.003), workplace setting (F = 5.50, *p* = 0.020), and years of service (F = 2.99, *p* = 0.019). Based on these findings, age group, job role, workplace setting, and years of service were retained and subsequently entered into the hierarchical regression analyses as background covariates. Other background characteristics, including sex, education level, marital status, and number of children, did not demonstrate statistically significant differences in self-actualization. Employment type, although not statistically significant in the preliminary group comparisons, was retained as a control variable in the regression models due to its theoretical relevance to occupational conditions. 

As detailed in the Methods section, these ANOVA results supported the inclusion of these covariates, which were selected based on both theoretical relevance and empirical associations. Therefore, post hoc comparisons were not conducted, as the purpose of this preliminary analysis was to inform covariate inclusion rather than to interpret specific group differences.

### Correlations among major constructs

Pearson correlation coefficients indicated that self-actualization was positively associated with individual health (*r* = 0.53, *p* < 0.001) and social support/environmental satisfaction (*r* = 0.53, *p* < 0.001). In contrast, significant negative correlations were observed with life stress (*r* = –0.37, *p* < 0.001), emotional exhaustion (*r* = –0.34, *p* < 0.001), and depersonalization (*r* = –0.40, *p* < 0.001). These correlations reflect concurrent associations among constructs and should not be interpreted as evidence of directional or causal relationships.

### Hierarchical regression analysis

Hierarchical regression results are presented in Table 5.

**Table 5:** Summary of hierarchical regression analysis for health-related factors of self-actualization (*N* = 328)

Variable	Model 1		Model 2		Model 3		Model 4		
	β	t	β	t	β	t	β	t	VIF
Age < 30	-.03	−0.52	-.04	−0.74	-.04	−0.86	-.04	−0.86	1.32
Age 41—50	.08	1.25	.01	0.09	.01	0.17	.002	0.03	1.63
Age ≥ 51	.18	2.55*	.05	0.76	.06	0.97	.05	0.87	1.92
Non-supervisory staff	-.15	−2.58*	-.04	−0.74	-.03	−0.53	-.03	−0.55	1.22
Health Bureau	-.12	−2.06*	-.07	−1.35	-.07	−1.45	-.07	−1.39	1.18
< 1 year of service	.16	2.27*	.13	2.13*	.12	2.07*	.13	2.13*	1.78
1—3 years of service	.01	0.18	.06	0.86	.06	0.85	.07	1.04	2.05
3—5 years of service	-.03	-.47	-.04	−0.64	-.04	−0.78	-.03	−0.61	1.51
5—10 years of service	.03	0.48	.03	0.49	.03	0.54	.04	0.75	1.55
Contract or temporary staff	.01	0.22	-.03	−0.65	-.03	−0.64	-.04	−0.76	1.32
Health and support resources									
Individual health and psychological well-being			.29	4.31***	.21	2.67**	.22	2.78**	3.25
Social Support and Environmental Satisfaction			.30	4.42***	.31	4.62***	.28	4.25***	2.20
Life Stress					-.14	−2.38*	-.15	−2.60*	1.76
Emotional Exhaustion							-.15	−2.21*	1.69
Depersonalization							-.20	−3.45**	1.32
R^2^	.09		.35		.36		.39		
Adj R^2^	.06		.32		.33		.36		
F	3.23**		14.06***		13.60***		13.03***		
△R^2^	.09		.26		.01		.03		
△F	3.23**		61.96***		5.66*		6.31**		

Standardized regression coefficients (β) are reported. Years of service were dummy coded, with ≥ 10 years of service as the reference group. Variance inflation factors (VIFs) indicated no evidence of multicollinearity among demographic variables

^*^p < *.05*

^**^p < *.01*

^***^p < *.001*

#### Model 1: demographic variables

Demographic factors accounted for 9% of the variance in self-actualization (R^2^ = 0.09, F = 3.23, *p* < 0.01). Having less than one year of service (β = 0.16, *p* < 0.05) and being aged 51 years and older (β = 0.18, *p* < 0.05) were significantly associated with higher levels of self-actualization. In contrast, non-supervisory roles (β = –0.15, *p* < 0.05) and being stationed at the Health Bureau (β = –0.12, *p* < 0.05) showed small but significant negative associations at this initial step. However, these associations should be interpreted with caution. In particular, the effect observed among early-career workers (< 1 year of service) may reflect short-term adjustment or “honeymoon” effects of new employment, or cohort-specific contextual influences, rather than stable developmental differences.

#### Model 2: health and social resources

The addition of individual health and social support/environmental satisfaction substantially increased the explained variance to 35% (R^2^ = 0.35, F = 14.06, *p* < 0.001), representing a large incremental contribution beyond Model 1 (ΔR^2^ = 0.26). Both variables demonstrated significant positive associations with self-actualization (β = 0.29 and β = 0.30, respectively; *p* < 0.001).

#### Model 3: life stress

Introducing life stress resulted in a modest increase in explained variance to 36% (R^2^ = 0.36, F = 13.60, *p* < 0.001), with a small incremental contribution relative to Model 2 (ΔR^2^ = 0.01). Life stress showed a significant negative association with self-actualization (β = –0.14, *p* < 0.05).

#### Model 4: full model

The final model accounted for 39% of the variance in self-actualization (R^2^ = 0.39, F = 13.03, *p* < 0.001), adding unique explanatory value beyond Model 3 (ΔR^2^ = 0.03). Compared with the substantial contribution of health and social resources in Model 2 (ΔR^2^ = 0.26), the additional variance explained by burnout-related variables was relatively modest, indicating a comparatively smaller incremental predictive contribution. Notably, most demographic associations observed in Model 1 were attenuated upon the inclusion of psychosocial predictors in Models 2 through 4, although having less than one year of service remained statistically significant in the final model. This pattern suggests that the apparent effects of demographic characteristics may be partly accounted for by underlying psychosocial resources and occupational strain factors, rather than age or tenure per se.

After adjustment for all other variables entered in the model, individual health (β = 0.22, *p* < 0.01) and social support/environmental satisfaction (β = 0.28, *p* < 0.001) remained significantly and positively associated with self-actualization. In contrast, life stress (β = –0.15, *p* < 0.05), emotional exhaustion (β = –0.15, *p* < 0.05), and depersonalization (β = –0.20, *p* < 0.01) remained significantly and negatively associated with self-actualization.

Consistent with the analytical strategy described in the Methods section, reduced personal accomplishment was not included in the regression models to avoid construct redundancy. Model diagnostics indicated no evidence of multicollinearity (VIF range = 1.18–3.25), and the Durbin–Watson statistic (1.74) supported residual independence, while residual plots confirmed assumptions of normality, linearity, and homoscedasticity.

### Summary

Overall, the hierarchical regression model explained 39% of the variance in self-actualization. Among the examined predictors, health-related resources—particularly individual health status and perceived social support/environmental satisfaction—accounted for the largest proportion of explained variance, whereas stress and burnout-related variables contributed comparatively smaller incremental effects. However, a substantial proportion of variance remains unexplained, suggesting the influence of additional unmeasured factors. All findings are interpreted within a cross-sectional framework and describe patterns of association rather than causal effects.

## Discussion

This study examined the biopsychosocial correlates of self-actualization among rural community healthcare workers (HCWs) using the Biopsychosocial Model (BPSM) as the primary organizing framework. The findings highlight that self-actualization—conceptualized here as active engagement in personal growth and meaning-making—is concurrently associated with the availability of health and social resources and negatively associated with psychological strain. Notably, health status and perceived social support accounted for the largest incremental proportion of explained variance (ΔR^2^ = 0.26), underscoring their salience within the biopsychosocial framework. Overall, the final model explained 39% of the variance in self-actualization. While this level of explained variance can be considered meaningful within the context of occupational health psychology (Cohen 1992; Schäfer & Schwarz 2019), the incremental contribution of burnout-related variables was comparatively modest (ΔR^2^ = 0.03), indicating that their additional predictive value should be interpreted with caution. Nevertheless, the fact that a substantial proportion of variance remains unexplained requires objective reflection; it suggests that other unmeasured factors—such as dispositional traits, organizational climate, or specific coping strategies—also play vital roles in shaping growth-oriented functioning.

### Biopsychosocial resources as foundations for self-actualization

Consistent with the BPSM as our overarching framework, the findings indicate that both individual health status and perceived social support are strongly and positively associated with higher levels of self-actualization. The substantial incremental variance explained by these factors suggests that physical vitality and supportive interpersonal environments function as important conditions associated with personal growth (Blount et al. 2021). In particular, the markedly larger incremental contribution of these resource-related variables compared to stress and burnout factors suggests that resource enhancement may play a more prominent role in growth-oriented functioning than stress reduction within this specific cohort. Operating as a supporting explanatory mechanism within the BPSM's social and psychological domains, this pattern aligns with conservation of resources theory, which posits that individuals require sufficient resource reserves before they can invest effort in higher-order growth processes.

Research on workplace interventions similarly indicates that enhancing psychological resources and strengthening supportive organizational structures is associated with improved fulfillment and engagement (Cherniack et al. 2024). The present findings underscore that community HCWs who report better physical health and stronger social support networks also report higher levels of perceived personal development and positive psychological functioning. However, given the cross-sectional design of the study, these associations must be interpreted cautiously. While health and social resources may enable growth-oriented functioning, it is equally plausible that reciprocal or reverse perceptual mechanisms are at play. Specifically, individuals with higher baseline self-actualization may possess a more positive cognitive appraisal style, leading them to perceive and report their health status and interpersonal support networks more favorably (Han et al. 2025).

### Psychological strain and burnout: distinct from lack of growth

Life stress and burnout emerged as significant negative correlates of self-actualization. Importantly, the present analysis focused specifically on the strain-related dimensions of burnout—emotional exhaustion and depersonalization—while deliberately excluding reduced personal accomplishment to avoid construct redundancy with the growth-oriented outcome of self-actualization. This analytic distinction reflects an effort to reduce conceptual overlap between psychological strain and growth-oriented functioning, although complete separation between these domains cannot be assumed.

The observed associations suggest that chronic emotional depletion and interpersonal detachment are closely linked to lower levels of perceived personal growth, a phenomenon that burnout theory helps explain as a specific psychological strain mechanism within the broader BPSM structure (Wuyts 2020). Rural community HCWs frequently encounter structural pressures, including blurred boundaries between professional and private life, broad role expectations, and limited organizational resources, all of which are associated with heightened psychological strain (Calhoun et al. 2022). By operationalizing life stress as a holistic indicator, the study captured the cumulative burden of these conditions.

Comparable patterns have been documented across international contexts, where rural HCWs exhibit elevated vulnerability to stress-related fatigue and emotional exhaustion (Moses et al. 2024; Zhang et al. 2024). Although emotional exhaustion and depersonalization remained statistically significant after accounting for health resources and life stress, their relatively small incremental contribution to explained variance suggests that these factors may play a more limited role in explaining variability in self-actualization compared to resource-related predictors. That emotional exhaustion and depersonalization remained significantly associated with self-actualization after accounting for health resources and life stress highlights the particular salience of relational and emotional depletion in relation to growth-oriented functioning. However, these findings do not imply that burnout causally impedes self-actualization, but rather that they co-occur within demanding occupational contexts.

### Demographic variability and professional development

Although initial group differences in self-actualization were observed across demographic variables, these effects were attenuated in the fully adjusted model, suggesting that demographic differences are likely explained by underlying psychosocial factors rather than direct effects of education or tenure. This attenuation is not merely a statistical artifact but also suggests that psychosocial resources may serve as more proximal correlates of growth-oriented functioning than demographic characteristics per se. Some demographic differences, particularly those related to age and years of service, may reflect competence accumulation processes, whereby professional knowledge, reflective capacity, and situational mastery develop over time. Furthermore, from a theoretical standpoint, these tenure-related differences may reflect occupational socialization processes and selection effects. It is plausible that workers who struggle to maintain positive psychological functioning or cope with severe role boundary demands in rural settings may be more likely to leave the workforce earlier, thereby leaving a more resilient and growth-oriented cohort among veteran staff (Muncey 1998).

At the same time, prior research suggests that prolonged exposure to resource-limited environments may also be associated with increased strain for some workers (Park & June 2022; Yang et al. 2023). Taken together, these findings suggest that education and tenure may be linked to both adaptive and challenging psychological experiences, underscoring the importance of tailored training, mentorship, and support strategies—particularly for early-career HCWs and those with fewer educational resources.

### Theoretical implications: distinguishing growth from well-being

A key theoretical contribution of this study lies in its application of the BPSM to map the correlates of positive psychological functioning among rural HCWs. While our initial conceptualization sought to differentiate self-actualization from general psychological well-being, we acknowledge that the operationalization used in this study likely captures shared variance within a broader eudaimonic well-being domain. In addition, several elements of the HPLP-based operationalization (e.g., meaning in life, personal strengths) conceptually overlap with constructs such as psychological capital and self-efficacy, suggesting that the measured construct may reflect a cluster of growth-oriented psychological resources rather than a fully distinct domain. Rather than representing a sharply distinct or ultimate developmental endpoint, self-actualization in this context is most appropriately situated within contemporary positive psychology literature, aligning closely with concepts such as flourishing, eudaimonic functioning, and psychological capital (Ploke et al. 2024).

While the HPLP self-actualization subscale shares conceptual space with constructs such as psychological capital (PsyCap) and self-efficacy, these constructs differ in emphasis: PsyCap and self-efficacy primarily capture individuals’ agentic capacity, confidence, and resilience in achieving task-specific goals, whereas self-actualization, as operationalized in this study, reflects a broader orientation toward meaning-making and the realization of personal potential. Clarifying these distinctions helps situate self-actualization as a related but not redundant construct within the broader landscape of workplace psychology.

Operating strictly as supporting theoretical lenses to inform the psychological outcome of the BPSM, humanistic and self-determination theories help explain why autonomy, competence, and meaning are core elements of this growth-oriented functioning (Maslow 1987; Ryan & Deci 2017; Wong 2011). The findings suggest that health and social support may function as a psychological safety net, whereas the absence of emotional exhaustion and depersonalization may be important for supporting the activation of growth-oriented functioning. This dual perspective extends occupational health literature by reframing HCW functioning not solely in terms of resilience or stress reduction, but in terms of conditions that support sustained psychological growth. Accordingly, the present findings should be interpreted as reflecting patterns within a broader eudaimonic well-being domain, rather than evidence of clearly separable psychological constructs.

## Conclusions, limitations, and implications

### Limitations of the study

#### Internal validity

The primary limitation concerns the timing of data collection. The dataset was collected in 2014 and therefore may not fully reflect more recent policy changes (such as the implementation of the "Long-Term Care 2.0" policy in Taiwan, which has shifted the structural demands of CHWs by expanding their caregiving network integration and administrative loads), organizational reforms, or the psychological effects of the COVID-19 pandemic. Nonetheless, the data provide a unique and theoretically informative historical baseline for understanding enduring patterns of stress, well-being, and work experiences in rural community health practice. Despite a decade of policy evolution, several structural challenges of rural health work—such as geographic isolation, blurred professional–personal boundaries, and intensive emotional labor—are likely to remain salient. Therefore, the psychosocial mechanisms identified in this pre-pandemic baseline may still hold theoretical and practical relevance for contemporary contexts. Given that the composition and core responsibilities of Taiwan’s community health workforce have remained relatively stable over the past decade, the findings retain interpretive value as a pre-pandemic reference point.

This baseline perspective is theoretically valuable, as it allows for the identification of relatively stable biopsychosocial associations prior to major systemic disruptions, thereby supporting future research in distinguishing enduring patterns from context-dependent effects (Pierce et al. 2020).

Second, because all measures relied on self-reported questionnaires, the results may be influenced by response tendencies such as social desirability. Self-report designs also raise the possibility of common method variance (CMV). To address this concern, procedural remedies were implemented (e.g., anonymity assurances and use of validated multi-item scales), and Harman’s single-factor test was conducted.

The results indicated that no single factor accounted for a majority of the variance. However, this test alone is insufficient to rule out CMV, and given the single-source, cross-sectional design, shared method variance may have partially influenced the observed associations.

Nevertheless, as with most cross-sectional behavioral research, shared method effects cannot be entirely ruled out (Podsakoff et al. 2003). Furthermore, the cross-sectional design strictly precludes any causal inferences; as noted in our discussion, the relationships between self-actualization, health resources, and burnout may very well be bidirectional. Future studies incorporating multi-source data or temporally separated measurements would further strengthen internal validity.

Finally, although the Biopsychosocial Model and burnout theory provided a useful conceptual framework, their specific applicability to rural community health contexts requires further empirical validation across diverse healthcare systems and cultural settings. Accordingly, theoretical interpretations should be viewed as contextually grounded rather than universally generalizable.

#### External validity

Participants were drawn exclusively from Hualien County, a rural and geographically distinctive region. As a result, the findings may not generalize to settings with different organizational structures, resource availability, or workforce compositions. In addition, contemporary healthcare reforms and broader societal changes underline the need for updated empirical studies to determine whether similar psychological patterns persist among today’s community health workers.

### Implications for practice and research

#### Implications for community health workers

Psychological well-being among community health workers may be supported through evidence-based strategies that enhance coping capacity, self-regulation, and personal meaning. Although demographic differences were attenuated in multivariable models, early-career workers descriptively showed comparatively lower self-actualization**;** therefore**,** targeted mentorship and onboarding programs may be particularly beneficial. Participation in structured stress-management programs, mindfulness-based interventions, and skill-building workshops has been shown to reduce emotional exhaustion and improve psychological functioning (Robertson et al. 2015). Specifically, to address the negative impact of depersonalization observed in this study, individual-level interventions should also focus on restoring interpersonal connection and managing emotional labor. Training in emotional boundary-setting and compassionate communication can help HCWs maintain professional empathy without succumbing to interpersonal detachment. Continuing education may also contribute to professional confidence, role identity, and opportunities for personal growth by reinforcing self-efficacy and intrinsic motivation—key contributors to growth-oriented psychological functioning (Ryan & Deci 2017).

#### Implications for health authorities

Health administrations may consider expanding resources in rural settings—including adequate staffing, equipment, and access to psychological support services—to help alleviate chronic occupational strain. Organizational interventions associated with improved well-being include structured supervision, participatory decision-making, and fostering psychologically safe climates for communication (Cooper & Cartwright 1994; West et al. 2016). To specifically combat the risk of depersonalization, organizations should foster a culture of collective care. Implementing structured reflective practices, such as Balint groups or regular peer-debriefing sessions, can provide safe, supportive spaces for HCWs to process the complex emotional toll of patient interactions and rebuild relational empathy. Considering the geographic isolation of rural regions like Hualien, these interventions—along with the aforementioned training in emotional boundary-setting—could be effectively implemented via telehealth or digital platforms. Such approaches may enhance accessibility and participation while minimizing additional travel burden for HCWs. Programs that promote peer support and resilience training may function as protective mechanisms against burnout. Policies supporting flexible scheduling, manageable caseloads, and opportunities for professional development may help cultivate environments conducive to sustained psychological functioning.

#### Implications for future research

Future research should incorporate diverse geographic regions, including urban–rural comparisons, to enhance generalizability and identify contextual moderators of psychological functioning. Longitudinal designs are particularly needed to clarify the temporal dynamics of stress, burnout, and self-actualization. The present 2014 dataset may serve as a theoretically meaningful benchmark for examining the psychological impact of subsequent social, economic, and policy changes, including the COVID-19 pandemic.

From a measurement perspective, future studies are encouraged to apply confirmatory factor analysis (CFA) to validate the factor structure of the adapted instruments in independent samples. Although exploratory factor analysis was appropriate for the current context-specific adaptation, CFA would allow more rigorous testing of construct validity and measurement invariance across different populations and settings.

Future research may also benefit from examining potential mediating or moderating factors—such as coping styles, self-efficacy, workplace social climate, or organizational justice—to further refine theoretical models of psychological functioning among community health workers.

## Conclusions

Guided by the Biopsychosocial Model, this study examined how individual health, psychological well-being, social support, life stress, and burnout are concurrently associated with self-actualization among rural community healthcare workers. Individual health and perceived social support emerged as the strongest positive correlates, whereas life stress, emotional exhaustion, and depersonalization showed significant negative associations with self-actualization. Consistent with the analytic strategy detailed in the Methods section, reduced personal accomplishment was excluded from the final model to avoid construct redundancy with the growth-oriented outcome of self-actualization. Together, these variables explained 39% of the variance in self-actualization, underscoring the value of integrating biological, psychological, and social perspectives when examining growth-oriented psychological functioning in rural health contexts.

Although the cross-sectional design precludes causal inference, the findings identify key psychosocial domains that warrant attention in organizational planning and future research. Aligning with contemporary positive psychology, self-actualization in this context is best understood as a vital facet of broader eudaimonic well-being. Rather than implying direct causal effects, the results highlight patterns of association suggesting that strengthening health resources, social support structures, and burnout prevention efforts may contribute to conditions supportive of personal growth and psychological functioning among rural community healthcare workers.

## Supplementary Information

Supplementary Material 1.

## Data Availability

The datasets analyzed during the current study are not publicly available due to institutional privacy restrictions but are available from the corresponding author on reasonable request and with permission from the Taipei City Hospital IRB (Approval No. TCHIRB-1030712-E).
