# Supplementary material for: Self-actualization and its psychosocial determinants among community health workers in rural Taiwan: a cross-sectional study
Source: BMC Psychol. 2026 May 13;14:996. doi: 10.1186/s40359-026-04709-w (PMC13339707; doi:10.1186/s40359-026-04709-w)
Supplement: Supplementary file 1 — Supplementary Material 1. [file 40359_2026_4709_MOESM1_ESM.docx]

**Part 1: Adapted WHOQOL-BREF Scale**

**Dimension 1: Individual Health and Psychological Well-being**

**WP1 (Original Item 1)** How would you rate your quality of life? *(1: Very poor; 2: Poor; 3: Neither poor nor good; 4: Good; 5: Very good)*

**WP2 (Original Item 2)** How satisfied are you with your health? *(1: Very dissatisfied; 2: Dissatisfied; 3: Neither satisfied nor dissatisfied; 4: Satisfied; 5: Very satisfied)*

**WP3 (Original Item 10)** Do you have enough energy for everyday life? *(1: Not at all; 2: A little; 3: A moderate amount; 4: Mostly; 5: Completely)*

**WP4 (Original Item 16)** How satisfied are you with your sleep? *(1: Very dissatisfied; 2: Dissatisfied; 3: Neither satisfied nor dissatisfied; 4: Satisfied; 5: Very satisfied)*

**WP5 (Original Item 8)** How safe do you feel in your daily life? *(1: Not at all; 2: A little; 3: A moderate amount; 4: Very much; 5: Extremely)*

**WP6 (Original Item 7)** How well are you able to concentrate? *(1: Not at all; 2: A little; 3: A moderate amount; 4: Very much; 5: Extremely)*

**WP7 (Original Item 15)** How well are you able to get around? *(1: Very poor; 2: Poor; 3: Neither poor nor good; 4: Good; 5: Very good)*

**WP8 (Original Item 5)** How much do you enjoy life? *(1: Not at all; 2: A little; 3: A moderate amount; 4: Very much; 5: An extreme amount)*

**WP9 (Original Item 17)** How satisfied are you with your ability to perform your daily living activities? *(1: Very dissatisfied; 2: Dissatisfied; 3: Neither satisfied nor dissatisfied; 4: Satisfied; 5: Very satisfied)*

**WP10 (Original Item 9)** How healthy is your physical environment? *(1: Not at all; 2: A little; 3: A moderate amount; 4: Very much; 5: Extremely)*

**WP11 (Original Item 14)** To what extent do you have the opportunity for leisure activities? *(1: Not at all; 2: A little; 3: A moderate amount; 4: Mostly; 5: Completely)*

**WP12 (Original Item 6)** To what extent do you feel your life to be meaningful? *(1: Not at all; 2: A little; 3: A moderate amount; 4: Very much; 5: An extreme amount)*

**WP13 (Original Item 11)** Are you able to accept your bodily appearance? *(1: Not at all; 2: A little; 3: A moderate amount; 4: Mostly; 5: Completely)*

**Dimension 2: Social Support and Environmental Satisfaction**

**WS1 (Original Item 22)** How satisfied are you with the support you get from your friends? *(1: Very dissatisfied; 2: Dissatisfied; 3: Neither satisfied nor dissatisfied; 4: Satisfied; 5: Very satisfied)*

**WS2 (Original Item 23)** How satisfied are you with the conditions of your living place? *(1: Very dissatisfied; 2: Dissatisfied; 3: Neither satisfied nor dissatisfied; 4: Satisfied; 5: Very satisfied)*

**WS3 (Original Item 24)** How satisfied are you with your access to health services? *(1: Very dissatisfied; 2: Dissatisfied; 3: Neither satisfied nor dissatisfied; 4: Satisfied; 5: Very satisfied)*

**WS4 (Original Item 20)** How satisfied are you with your personal relationships? *(1: Very dissatisfied; 2: Dissatisfied; 3: Neither satisfied nor dissatisfied; 4: Satisfied; 5: Very satisfied)*

**WS5 (Original Item 19)** How satisfied are you with yourself? *(1: Very dissatisfied; 2: Dissatisfied; 3: Neither satisfied nor dissatisfied; 4: Satisfied; 5: Very satisfied)*

**WS6 (Original Item 28)** How often do you have the opportunity to eat the food that you like? *(1: Never; 2: Seldom; 3: Quite often; 4: Very often; 5: Always)*

**WS7 (Original Item 18)** How satisfied are you with your capacity for work? *(1: Very dissatisfied; 2: Dissatisfied; 3: Neither satisfied nor dissatisfied; 4: Satisfied; 5: Very satisfied)*

**WS8 (Original Item 25)** How satisfied are you with your transport? *(1: Very dissatisfied; 2: Dissatisfied; 3: Neither satisfied nor dissatisfied; 4: Satisfied; 5: Very satisfied)*

**WS9 (Original Item 21)** How satisfied are you with your sex life? *(1: Very dissatisfied; 2: Dissatisfied; 3: Neither satisfied nor dissatisfied; 4: Satisfied; 5: Very satisfied)*

**Part 2: Adapted Life Stress Questionnaire (CHQ-12)**

**Description:** The items below are from the Chinese Health Questionnaire (CHQ-12). After item analysis, items 7 and 10 were removed. The remaining 10 items constitute the "Life Stress" dimension.

**Response Scale:** Respondents are asked how they have felt **over the past few weeks**. *(1: Not at all; 2: Same as usual; 3: Rather more than usual; 4: Much more than usual)*

**Dimension: Life Stress**

**C1 (Original Item 1)** Have you lately had headaches or a feeling of pressure in your head?

**C2 (Original Item 2)** Have you lately had palpitations or heart thumping and worried that you might have heart disease?

**C3 (Original Item 3)** Have you lately had a feeling of discomfort or pressure in your chest?

**C4 (Original Item 4)** Have you lately had shaking or numbness in your hands or feet?

**C5 (Original Item 5)** Have you lately been sleeping badly?

**C6 (Original Item 6)** Have you lately felt that many things are a burden to you?

**C8 (Original Item 8)** Have you lately lost confidence in yourself?

**C9 (Original Item 9)** Have you lately felt nervous and strung-up (tense)?

**C11 (Original Item 11)** Have you lately felt that your family or friends are worrying you?

**C12 (Original Item 12)** Have you lately felt that life is entirely hopeless?

**Part 3: Adapted Workplace Burnout Scale**

**Description:** The scale was adapted from the Copenhagen Burnout Inventory (CBI) and localized for the study context. The original pool contained 21 items. Following item analysis, Item 8 and Item 21 were removed. The final scale consists of **19 items** categorized into three dimensions.

**Response Scale:** (1: Strongly Disagree; 2: Disagree; 3: Agree; 4: Strongly Agree)

**Dimension 1: Emotional Exhaustion (9 items)**

**WE1 (Original Q1)** Do you often feel tired?

**WE2 (Original Q2)** Do you often feel physically exhausted (drained of energy)?

**WE3 (Original Q3)** Do you often feel emotionally exhausted?

**WE4 (Original Q4)** Do you often think: “I can’t take it anymore”?

**WE5 (Original Q5)** Do you often feel weak, as if you are about to get sick?

**WE6 (Original Q6)** Is your work emotionally exhausting?

**WE7 (Original Q7)** Does your work frustrate you?

**WE8 (Original Q9)** Are you exhausted in the morning at the thought of another day at work?

**WE9 (Original Q10)** Do you feel that every working hour is tiring for you?

**Dimension 2: Depersonalization (6 items)**

**WD1 (Original Q11)** Do you find it hard to interact with clients?

**WD2 (Original Q12)** Does it drain your energy to work with clients?

**WD3 (Original Q13)** Do you wish to reduce the amount of time you spend in contact with clients?

**WD4 (Original Q14)** Do you feel annoyed by clients?

**WD5 (Original Q15)** Do you feel that you give more than you get back from clients?

**WD6 (Original Q16)** Do you have thoughts of wanting to get rid of clients quickly?

**Dimension 3: Reduced Personal Accomplishment (4 items)**

**WL1 (Original Q17)** Do you start thinking about work as soon as you wake up in the morning?

**WL2 (Original Q18)** Do you still think about work after you get home?

**WL3 (Original Q19)** Do you still think about work when you go to bed?

**WL4 (Original Q20)** Do you sacrifice other activities for the sake of your work?

**Part 4: Adapted Health-Promoting Lifestyle Profile (Self-Actualization Subscale)**

**Description:** The items are derived from the Health-Promoting Lifestyle Profile (HPLP). For this study, the "Self-actualization" subscale (8 items) was selected. Based on item analysis and factor analysis, all 8 items were retained.

**Response Scale:** *(1: Never; 2: Sometimes; 3: Often; 4: Always)*

**Dimension: Self-actualization**

**H1 (Original Item 14)** I can appreciate myself.

**H2 (Original Item 15)** I am confident and optimistic about life.

**H3 (Original Item 16)** I feel that I am growing and changing in a positive direction.

**H4 (Original Item 17)** I know where my strengths and weaknesses lie.

**H5 (Original Item 18)** I know what is important to me in life.

**H6 (Original Item 19)** I value my own achievements.

**H7 (Original Item 20)** I find every day to be full of interest and challenge.

**H8 (Original Item 21)** I feel that my life has meaning.
